# Fungal-mediated soil aggregation as a mechanism for carbon stabilization

**DOI:** 10.1093/ismejo/wraf074

**Published:** 2025-04-18

**Authors:** Steven P C de Goede, S Emilia Hannula, Boris Jansen, Elly Morriën

**Affiliations:** Department of Ecosystem and Landscape Dynamics, Institute of Biodiversity and Ecosystem Dynamics (IBED-ELD), University of Amsterdam, PO Box 94240, 1090 GE Amsterdam, The Netherlands; Department of Terrestrial Ecology, Netherlands Institute of Ecology (NIOO-KNAW), PO Box 50, 6700 AB Wageningen, The Netherlands; Department of Terrestrial Ecology, Netherlands Institute of Ecology (NIOO-KNAW), PO Box 50, 6700 AB Wageningen, The Netherlands; Department of Environmental Biology, Institute of Environmental Sciences (CML), Leiden University, Einsteinweg 2, 2333 CC Leiden, The Netherlands; Department of Ecosystem and Landscape Dynamics, Institute of Biodiversity and Ecosystem Dynamics (IBED-ELD), University of Amsterdam, PO Box 94240, 1090 GE Amsterdam, The Netherlands; Department of Ecosystem and Landscape Dynamics, Institute of Biodiversity and Ecosystem Dynamics (IBED-ELD), University of Amsterdam, PO Box 94240, 1090 GE Amsterdam, The Netherlands; Department of Terrestrial Ecology, Netherlands Institute of Ecology (NIOO-KNAW), PO Box 50, 6700 AB Wageningen, The Netherlands

**Keywords:** arbuscular mycorrhizal fungi (AMF), plant organic matter input, plant roots, saprotrophic fungi (SF), soil aggregation, soil organic carbon, soil C models, rhizosphere

## Abstract

Soils can potentially be turned into net carbon sinks for atmospheric carbon to offset anthropogenic greenhouse gas emissions. Occlusion of soil organic carbon in soil aggregates is a key mechanism, which temporarily protects it from decomposition by soil organisms. Filamentous fungi are recognized for their positive role in the formation and stabilization of aggregates. In this review, we assess the current knowledge of the contribution of fungi to soil aggregation and set a new research agenda to quantify fungal-mediated aggregation across different climates and soils. Our review highlights three main knowledge gaps: (1) the lack of quantitative data and mechanistic understanding of aggregate turnover under field conditions, (2) lack of data on the biochemical and biological mechanisms by which filamentous fungi influence soil aggregation, and (3) uncharacterized contribution of soil fungi across environments. Adopting a trait-based approach to increase the level of mechanistic understanding between fungal diversity and soil structure seems promising, but will need additional experiments in which fungal diversity is manipulated by either removal through sieving or dilution, or addition through using synthetic communities of cultured fungi. We stress the importance of integrating ecological and physicochemical perspectives for accurate modelling of soil aggregation and soil organic carbon cycling, which is needed to successfully predict the effects of land management strategies.

## Introduction

One of the key ecosystem functions of soils is the capacity to store organic carbon [1]. With an estimated 2400 Gt carbon, soils make up the largest terrestrial carbon pool, storing three times more carbon than the atmosphere [2, 3]. Anthropogenic managed systems have been turned from carbon sinks into carbon sources and need to be converted back into net carbon sinks for atmospheric carbon and then to reducing anthropogenic greenhouse gas emissions (CO_2_, CH_4_) causing climate change [4, 5]. To increase soil organic carbon (SOC) stocks in, for instance, agricultural and grassland ecosystems, management practices adapted to local conditions (climate and soil type) should be implemented [6]. This requires a thorough understanding of the underlying biotic mechanisms and their interactions with the abiotic environment that drive SOC storage in a wide variety of circumstances [7].

**Figure 1:**
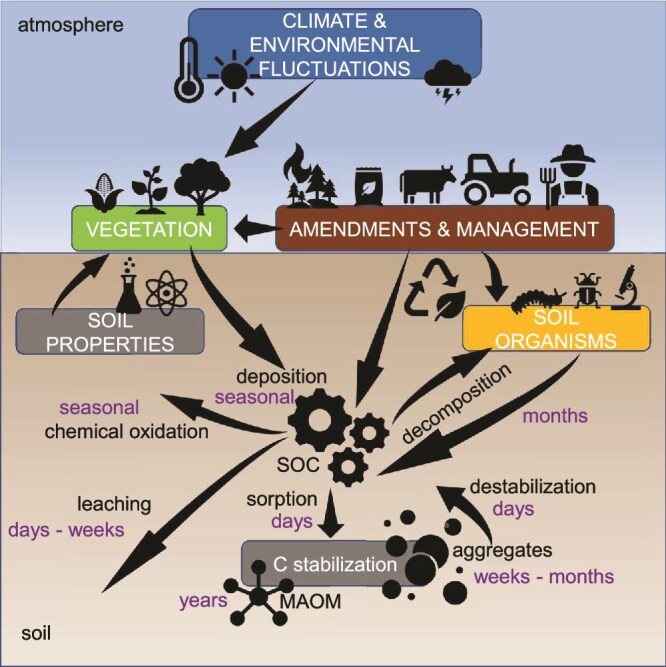
Overview of soil organic carbon (SOC) dynamics, its controlling factors, and their interactive relationships. The controlling factors can be simplified in five groups indicated as boxes: Climate and environmental fluctuation, soil properties, vegetation, amendments, and organisms including fungi, which are interconnected. The three-stepped process of SOC dynamics: (i) deposition, (ii) sorption which leads to stabilization, and (iii) destabilization. Relevant processes per factor group are indicated with arrows. Combinations of soil properties, vegetation, and amendments determine the decomposition of inputs and recycling of nutrients. For deposition the most important sources are given: plant residues, exudates, amendments and decomposition products, and necromass of organisms. For stabilization the key mechanisms are sorption into mineral-associated organic matter (MAOM) and occlusion into aggregates, and for destabilization the key mechanisms are decomposition, chemical oxidation, and leaching. For all processes, we indicated the time frames in which the process takes place or duration of the stabilization in terms of the MAOM and aggregate pool. They are drawn based on a best estimated guess from wider literature and what we encounter in our own experiments.

In the last decade, the soil science community has seen a paradigm shift regarding the mechanisms involved in SOC stabilization [8–11]. Previously, it was thought the molecular composition of soil organic matter (SOM) controlled its decomposition, with large, complex molecules (“humic substances”) being slowest to be turned over. However, the current view is that SOC stabilization is primarily determined by biological and environmental controls (Fig. 1). The interaction of the environment with the biological component determines the ability of soil organisms to decompose SOM [8, 9]. Figure 1 depicts the five key factors and processes involved in SOC stabilization, namely climate and environment, soil management, vegetation, soil properties, and soil organisms. A key mechanism is the adsorption of SOM to soil mineral surfaces (mineral-associated organic matter, MAOM) and occlusion in soil aggregates (Fig. 1; C stabilization), limiting the accessibility of organic carbon to the microbial community [12–14]. This is relatively well-studied from soil physical and chemical perspectives (soil properties) [15], but the soil biological community (soil organisms) has been treated as a black box for a long time [16, 17]. Nonetheless, soil (micro-)organisms are one of the main drivers of SOC dynamics [1, 18], and hence, a good mechanistic understanding of their role is required to successfully apply measures (soil management) to increase SOC storage and to improve mechanistic modelling enabling realistic predictions from soil C models.

Fungi are key organisms within soil microbial communities, especially responsible for carbon cycling and transformations of organic material [19, 20]. Recent studies found that fungi are responsible for sequestration and stabilization of up to 75% of plant-derived carbon in natural grasslands (based on stable isotope tracing of plant-derived rhizodeposits), whereas they only represent ca. 35% of total microbial biomass [21–23]. Apart from SOC deposition in soil, fungi can also help stabilizing organic carbon in soils (Fig. 1) by contributing to the formation and stabilization of soil aggregates [24, 25]. Hyphal networks of fungi, similar to plant roots, often referred to as “sticky string bag”, can potentially translocate and enmesh soil particles and organic matter and stabilize these in aggregates [26–28]. However, the relative importance that soil fungi have on aggregation in comparison with other biotic (bacterial mucilage contribution) and abiotic factors (soil texture, pH) is still unknown [29].

Bacteria also produce sticky substances as mucilage or chemical communication byproducts. These extracellular polymeric substances (EPS) also contribute to aggregate stability [30]. Furthermore, the soil texture (ratio of sand, silt, and clay) determines the natural adherences of primary minerals and also affects the community assembly in the given soil. Quantifying the relative importance of soil fungal processes compared to the abovementioned factors is crucial to understand soil aggregation and SOC (turnover) models and to determine under which circumstances and how soil fungi should be considered in the implementation of management practices targeting increased SOC stabilization.

The importance of occlusion in aggregates as an alternative mechanism for SOC protection has not gained much attention in scientific discourse due to current focus on the microbial carbon pump [31–34] and mineral association of microbial residues [18, 35, 36]. Likely, all three mechanisms are important for SOC stabilization and deserve a similar amount of attention. In fact, these mechanisms provide much of the MAOM and particulate organic matter (POM) that are key candidates to get occluded in soil aggregates and thereby become less susceptible for mineralization when the soil column receives fresh carbon inputs from the vegetation as root exudates and root and shoot litter [37–39]. Fungi are able to break down complex carbon molecules but often require fresh carbon inputs to do so. Due to their hyphal growth, they also contribute significantly to aggregate stability [40].

In this literature review, we assess the current knowledge on the contribution of soil fungi to soil aggregation. We specifically focus on croplands and natural and agricultural grassland ecosystems as they cover a vast area of the world, are often degraded, and can be managed and hence have a great potential for improved SOC storage [4, 5, 41]. The following three research areas will be addressed with focus on the role of saprotrophic and mycorrhizal fungi:

1. Soil fungal contribution to the stabilization of SOC.
2. Inclusion of soil fungi in quantitative soil aggregation models across environments.
3. Knowledge gaps in the role of soil fungi in carbon storage through aggregate formation.

## Soil organic carbon stabilization and soil aggregation

### Controlling factors of soil organic carbon dynamics and soil aggregation

**Figure 2:**
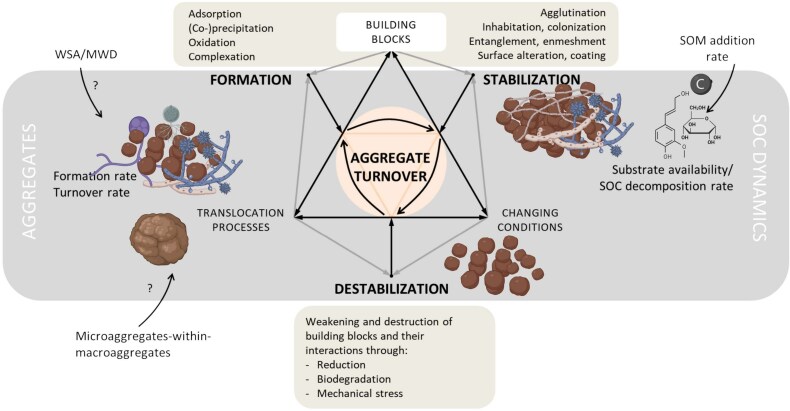
Conceptual model of the continuum of soil aggregation, with underlying mechanisms within the processes deposition/formation, stabilization, and destabilization related to Fig. 1. The formation, stabilization, and destabilization of macroaggregates and microaggregates in soil happen simultaneously. Interactions between the mechanisms that control the available (composite) building blocks, the active translocation processes, and potentially changing (soil) conditions which all underlie the formation and turnover of soil aggregates. The nature and associated processes of each of these three pillars of aggregation depends on the other two and the current state of aggregation. The most important abiotic and biotic processes for formation and stabilization and destabilization are given. WSA/MWD, SOM addition rate, and/or microaggregates-within-macroaggregates may be used as indicators for certain aspects of soil aggregation which are for a large part highly influenced by soil microbes by which fungi are the only ones that contribute both with exudates and physical enmeshment. SOC: soil organic carbon; SOM: soil organic matter; WSA: water-stable aggregates; MWD: mean weight diameter (of aggregates). Losing fungi by physical disturbances such as ploughing will enhance the destabilization of (macro)aggregates.

Soil aggregates are composite structures made of a large number of different building blocks, such as primary minerals, phyllosilicates, metal hydroxides, and organic matter [42]. These building units of biological, organic, or inorganic soil components can serve as formation nucleus, gluing agent, or cementing agent. The latter two are, respectively, of organic and inorganic origin and bring together other building units. Soil aggregation is a continuous process during which aggregates are simultaneously formed, stabilized, and broken down as a consequence of various chemical, physical, and biological processes in which fungi play an important role (Fig. 2).

In most soils where SOM is the prevailing binding agent, aggregation can be described by the hierarchy concept originally proposed by Tisdall and Oades [43] and modified by Oades [27] and Six et al. [44]. Recently, aggregates are being assessed on the soil surface using trained convolution neural networks to recognize different sizes and classes of aggregates based on stereo images [45]. Therefore, not all aggregates are equivalent. At different hierarchal levels, different mechanisms and binding agents will act during formation and disintegration of aggregates. Smaller aggregates, also called microaggregates usually in the ranges 20–250 μm or 53–250 μm, are found to be more stable than the larger aggregates as they consist of strong associations between more persistent and smaller organic matter particles, phyllosilicates, (hydr)oxides of Fe, Al, Mn, and carbonates and hence can withstand greater disruptive forces. In contrast, macroaggregates (>250 μm) are made up of microaggregates and larger soil particles bound together by weaker, more temporary, and transient binding agents such as recently deposited organic materials, plant roots, and fungal hyphae. Macroaggregates, especially in agricultural soils, are found to be mainly formed around largely undecomposed POM (usually >50–250 μm) and small plant roots that serve as precipitation nuclei [46].

Microorganisms and roots growing in and around the aggregate help form and stabilize the composite structure [24, 47–49]. New microaggregates are said to be formed within macroaggregates [15, 26, 50], where contact between various building blocks will be greatest (Fig. 2), but direct evidence for this in natural soils is missing. Upon destabilization of macroaggregates after organic matter decomposition or physical disruption, microaggregates are released and can be incorporated in newly formed macroaggregates [44] (Fig. 2). To maintain a larger number of stable macroaggregates, a continuous input of fresh POM is required to act as nucleus and stimulate microbial activity needed for formation and stabilization (Fig. 2) [51]. The aggregate hierarchy does not, however, apply to all soil types, such as those containing large concentrations of metal oxides (volcanic soils) as they form large Al-humus complexes that would already classify in aggregate size classes or sandy soils with low amounts of SOM [26, 52], as they might consist of just loose sand particles.

Lavallee et al. [46] proposed to entirely disregard aggregate fractions (>50 μm) within SOC stabilization research related to global changes and global SOC stocks, and urges to focus on MAOM and POM. MAOM consists of singular compounds or microscopic organic fragments chemically associated with mineral surfaces (organo-mineral associations), forming important building blocks of microaggregates [53]. MAOM is most likely growing from the dissolved organic matter (DOM) fraction in water-filled pores surrounding these microaggregates [54]. DOM consists of short polymers like plant and microbial metabolites. DOM is most likely to bind to the mineral fractions of the soil column leading to MAOM [54]. An increased soil porosity and pore connectivity can lead to a more efficient preprocessing of new DOM by microbes such as fungi (Table 1), which then binds to the MAOM fraction via cation bridging [55]. In turn, microbial activity affects soil porosity and aggregate turnover [56, 57].

**Table 1:** Mechanisms of fungal-mediated soil aggregation and their associated fungal functional traits. To increase our mechanistic understanding, it may be important to disentangle the fungal effects on the three process components of aggregation: formation (F), stabilization (S), and destabilization (D). Each proposed mechanism (also Fig. 3) can be linked to a fungal trait complex, and examples of (proposed) underlying measurable true traits are listed with unit where possible.

**F**	**S**	**D**	**Mechanism**	**Trait complex/description**	**Examples of proxies/measured traits**
x			Alignment and compression	Fungal-mediated particle movement	Hyphal density related to porosity [124] Particles moved per time unit [124]
x			Adhesion and orientation of particles	Hyphal surface properties	Hyphal surface charge per length [124] *Soil mass attached per hyphal length* *Biochemistry/composition hyphal wall*
x	x		Enmeshment and entanglement	Mycelial architecture/morphology and extension (rate)	Hyphal length (density) [m/g soil] [89, 124] Specific hyphal length [e.g. cm/mg root] [124] Biomass density [mg/cm^2^] [85] Hyphal density [124] [e.g. cm/cm^3^ soil] Hyphal surface area [μm^2^] [85] Branching angle [°] [85] Hyphal diameter [μm] [85] Internodal length [μm] [85] Mycelial complexity [-] [85] Lacunarity/gappiness [-] [85] (Radial) colony extension rate [μm/h] [85] Root-hyphae distance/max. extension [124] Intensity of root-hyphae linkages [124]
(x)	x		Gluing agents and surface sealing	Quality and quantity of produced fungal exudates and fungal necromass	Glomalin [GRSP fraction, mg/g soil] [162] *Hydrophobins* Exudate quality [C:N ratio] [124]
(x)	x		Soil water regime alteration	Mediation of wet-dry cycles, drying around hyphae	Hyphal water transport [124] *Soil water potential around hyphae*
	x		Increasing surface hydrophobicity	Water repellency of aggregates and hyphae	Water drop penetration time of aggregate surfaces [s] [104] Ethanol molarity drop test of hyphal surfaces [% molarity] [85]
(x)	(x)		Hyphal durability	Longevity, resistance and resilience against disturbances	Tensile strength [e.g. kPa [89], hyphal diameter, wall thickness [29] ] Repair capacity [hyphal growth after disturbance, e.g. μm/h, cm/cm^3^] [124] Palatability [# collembola faecal pellets] Decomposability [*e.g. melanin content, C:N ratio*] [163] Life span [e.g. mortality hyphae/g soil] [124]
(x)	(x)	x	Decomposition with degradative enzymes	Repertoire and activity of decomposition enzymes	Laccase [activity/g dried hyphal mass] [85, 148] Cellobiohydrolase [activity/g] [85, 148] Leucine aminopeptidase [activity/g] [85, 148] Acid phosphatase [activity/g] [85, 148]
		x	Penetration and tunnelling	Aggregate physical disruption by hyphae	*Intra-aggregate porosity* *Intra-aggregate colonization rate*

GRSP: glomalin-related soil protein; Traits/proxies in italic are not mentioned within this context in literature.

POM binds on and becomes occluded in aggregates and consists of long polymers derived from microbial necromass and decaying litter fragments. Litter inputs (mainly from decaying roots in grasslands and croplands) consist of high molecular weight polymeric material such as tannin, lignin, suberin, and cutin, which can be used as carbon source by decomposer microbes [58]. Therefore, microbial necromass fractions and long polymers are the most dominant molecular POM form inside aggregates, whereas plant and microbial metabolites are more likely to occur as DOM in water-filled pores before they end up as MAOM on binding sites [59]. Also fungi might be able to directly transport DOM and POM particles that stick to their hyphae through adhesion (Table 1 in Ref. [60]).

The existence of soil aggregates has been debated extensively in soil science, resulting in two seemingly opposing views: the aggregate (or solid phase) perspective vs. the pore space (or architectural) perspective [61, 62]. Aggregates are difficult to study within intact soil columns, even with advanced techniques as 3D X-ray tomography [63] and have been dismissed as perhaps being merely an artefact of soil sampling or tillage. When studying soil microbes and fauna (such as nematodes and protists), pore spaces are very relevant as their size, connectivity, and nature (whether they are water-filled or air-filled) defines the micro-habitat for these organisms [64, 65]. Many soil researchers see the two perspectives not as mutually exclusive, rather being two sides of the same coin [61]. Indeed, the composition of soil constituents and many of the processes summarized in Fig. 2 also determine pore architecture. Integrating the perspectives is likely the way forward, with more attention for quantifying pore spaces as methods evolve and become more accessible [66, 67]. Research thus far on the role of fungi in shaping soil structure seems to have focussed (but not exclusively) on aggregation and not pore spaces, especially in relation to SOC stabilization. Nevertheless, we expect similar fungal-mediated mechanisms to affect the organization of both particles and pores, thereby altering the accessibility of decomposers to organic substrates. Aggregates offer limited protection from decomposition on decadal-to-centennial timescales compared to MAOM as this is better protected against degradation by chemical bonds. But occluded organic matter particles can, although they are less stable, accumulate indefinitely [68]. Including aggregates in general, and aggregate stability specifically, is needed to properly understand POM-MAOM and SOC dynamics as a whole. Therefore, we encourage that studies focusing on MAOM fractions also report the occluded particulate organic matter fractions, as this will provide knowledge to evaluate the models of SOC dynamics. This is especially crucial for studies also analysing the abundance and diversity of specific soil organisms (fungi and bacteria) and faunal groups (collembola, mites, nematodes, and earthworms) [69, 70] as this will enable us to evaluate the role of soil biota in aggregate stability and SOC dynamics.

## Soil fungi and their potential effect on soil aggregation

### Soil fungal diversity

Among the soil organisms, fungi are critically important for various soil functions such as biogeochemical cycling of nutrients [19, 71]. Fungi are hugely diverse with an estimate of up to 6 million species globally [72]. Within this very large diversity important distinctions can be made based on life cycle and morphological characteristics that will be relevant for soil aggregation. Some fungi exist primarily as (clusters of) single cells (yeasts), whereas others grow as a mycelium, branching, root-like structures consisting of fungal hyphae. As this filamentous growth can extend over a larger area, the fungus will have a more stable supply of water and nutrients, whereas single-celled fungi rely on local nutrient availability. Studies about the effects of unicellular fungi on soil aggregation are scarce, showing variable effects on aggregation ranging from positive to negative [73] and will not be discussed here.

Filamentous fungi potentially affecting soil aggregation can be split in saprotrophic, pathogenic, endophytic, and mycorrhizal fungi [74]. Saprotrophic fungi (SF) roam “freely” in the soil in search of organic materials to break down using extracellular enzymes and eventually ingest. Conversely, mycorrhizal fungi form symbiotic relationships with plants and deliver vital and often-limiting nutrients, especially nitrogen and phosphorus, to their hosts in return for sugars and fatty acids [19, 75]. On top of this, all fungi can transport nutrients and water [76–78] over large distances in soils and serve as natural highways for other microbes and fauna in soil [79–81]. Here, we will mostly focus on arbuscular mycorrhizal fungi (AMF; Glomeromycotina) as these are most important type of mycorrhizal fungi in agricultural and grassland ecosystems [19, 82]. Moreover, AMF are far more intensely studied in relation to soil aggregation than the other mycelial fungal functional guilds [24, 29].

### Fungal mechanisms affecting soil aggregation

The importance of filamentous fungi in soil aggregation has been recognized for decades [47, 48, 73], although hyphal forming bacteria (Actinomycetes) are also important contributors to soil aggregation [73]. Effects of filamentous fungi on soil aggregation have been estimated to be similar or higher than that of soil bacteria and much larger than that of soil animals [41], making them a major player in soil aggregation. The contribution of bacteria vs. fungi in soil aggregation is likely to vary between ecosystems and reflect the relative contributions of these organisms to the total soil biomass. The fungi to bacteria ratio (F/B) decreased as follows: forests > croplands > grasslands, with soils in colder climates showing greater F/B ratios in croplands and forests. On top of that, soil texture, SOC, and nitrogen were shown to directly drive bacterial and fungal biomass [83]. Furthermore, the composition of the soil fungal community is likely to affect aggregation as different phyla have different abilities to aggregate soils [41]. Leifheit et al. [84] found a positive overall effect of AMF inoculation on soil aggregation (i.e. increased (macro)aggregate stability) in a meta-analysis covering studies between 1986 and 2012. SF, and especially Ascomycota and Mucoromycotina, generally also positively contribute to soil aggregation, but fewer quantitative studies are available [25, 73, 85]. Filamentous fungi are found to mostly affect the formation and stabilization of water-stable macroaggregates, and not free (unaggregated) microaggregates, with limited attention given to the formation of microaggregates within macroaggregates [17, 48, 86].

**Figure 3:**
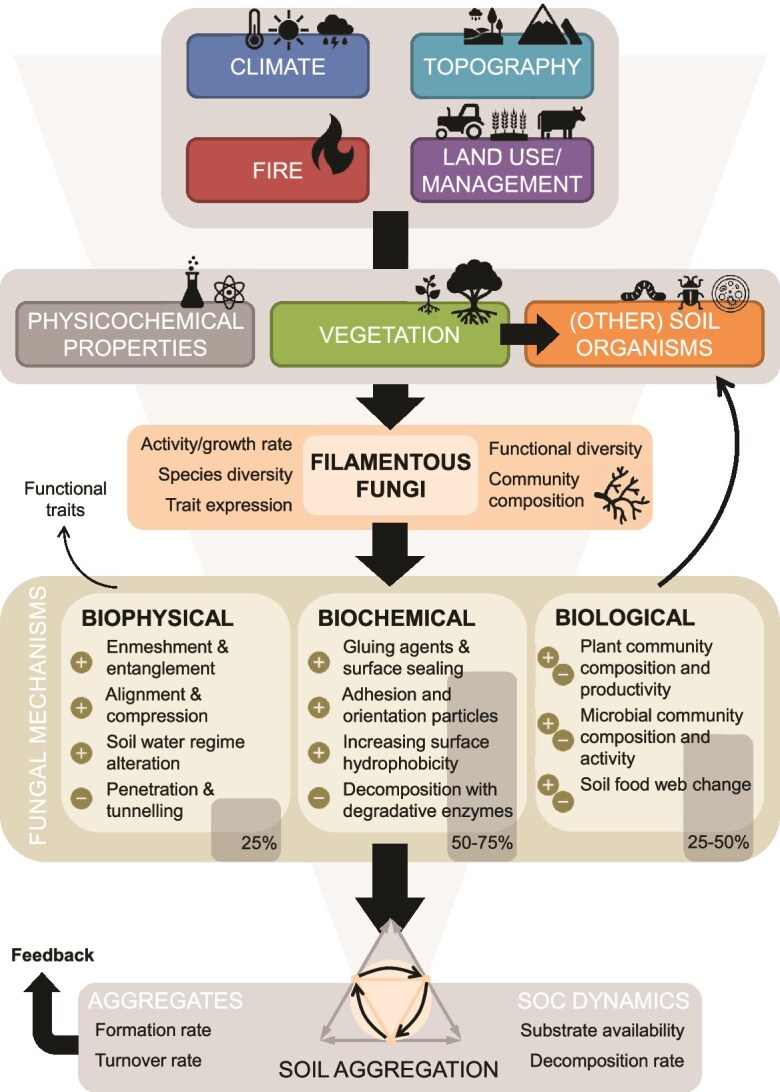
Overview of the relationship between filamentous fungi and soil aggregation, and the mechanisms needed to study and quantify this relationship. From top to bottom roughly ordered by decreasing scale. The fungal mechanisms can be converted to functional traits to quantify their role in the formation, stabilization, and destabilization of soil aggregates (see also Table 1). Estimated percentages based on literature show how much each fungal mechanism contributes to aggregate stability is indicated in the figure. This figure combines the controlling factors of Fig. 1 with the mechanisms of Fig. 2. The relative effect of the fungal mechanisms indicated are percentages divided within fungal effects on soil aggregation. The overall contribution of fungi to soil aggregation relative to all other factors is heavily context dependent.

Despite many studies and narrative reviews covering the subject, the exact mechanisms through which filamentous fungi affect soil aggregation, as well as their overall relative importance, are not fully resolved [29] as many experiments are done in very artificial conditions lacking insight on interaction effects of soil fauna or different environmental conditions. It is difficult to disentangle the effects of all possible abiotic and biotic factors and mechanisms from those mediated by soil fungi, or to separate the interacting fungal mechanisms from each other. Figure 3 shows the potential (*biophysical*, *biochemical*, and *biological*) mechanisms by which fungi can aid in soil aggregate formation (SAF) ultimately leading to SOC stabilization. However, at some point in time all formed aggregates destabilize due to changing conditions (land management practices) after which aggregates fall apart and the formation process starts again, called aggregate turnover (Fig. 3).

### Biophysical mechanisms

Due to the root-like growth and architecture of the mycelium, fungi are expected to affect soil structure analogous to the physical action of roots. Entanglement (or enmeshment) of soil particles and aggregates by fungal hyphae is often mentioned as one of the key mechanisms (Fig. 3), but surprisingly, little evidence is available for this process. Figure 3 summarizes and relates factors, processes, and mechanisms from Figs 1 and 2 into one conceptual scheme. Some correlational evidence linked increasing length of the mycelial network to more water-stable (macro)aggregates, but the exact underlying mechanism(s) remains unclear [48, 87, 88]. Observations by scanning electron microscopy (SEM) have provided the most direct evidence for hyphal entanglement [17, 29, 87–89], although this is mostly for SF and still does not prove that this particular physical action significantly changes aggregate formation or turnover. Degens [48] also mentions cross-linkage as a process separate to enmeshment, mainly applying to soils having coarser sand and large number of aggregates >150 μm.

As hyphae grow, they push and reposition soil particles, leading to local compression and alignment which may enhance aggregate formation (Fig. 3). However, this specific mechanism has never been experimentally studied in fungi, and evidence is limited to few SEM observations [90, 91]. Studies have shown that mycorrhizal fungi in conjunction with their host plants can locally alter the water regime, moving water and nutrients from the soil to the plant and vice versa over great distances [92, 93]. This drying and wetting can affect the formation and stabilization of aggregates via physical and chemical processes [94]. However, Védère et al. [28] shows only weak relations between soil moisture content and aggregation in the detritusphere of artificial packed columns, compared to the biotic effects on aggregation.

### Biochemical mechanisms

Fungi produce numerous compounds which are excreted by the hyphae while alive or released during their decomposition, and these form the basis for the biochemical mechanisms. Fungal products have since long been suggested to act as gluing agents between the various building blocks of soil aggregates (Fig. 3), or as surface sealers of particles and aggregates [17, 28, 90]. Evidence for this mechanism comes from SF in experimental settings [88, 95, 96], leaving open the question to what degree this applies to AMF and natural soils. Studying fungal exudates in soils is challenging due to the large variety of organic compounds and possible origins, and the difficulty of analytically separating them. Research has focused on fungus-derived polysaccharides and (glyco)proteins, sometimes referred to as hyphal exudates [97, 98]. The presence of glue-like compounds on hyphae can cause adhesion of particles [99], which will interact with the enmeshment mechanism to form the “sticky string-bag” of hyphae [28]. Spatially resolved chemical characterization of microbial–mineral interfaces is crucial for an increased understanding of overall carbon cycling in soil and the role of fungi therein.

Hydrophobins, small molecular weight (5–20 kDa) proteins, containing amino acid residues [100] produced by filamentous fungi, have been suggested to be among the most prominent compounds with several functions ascribed to them, mostly relating to alteration of hyphal surface properties and attachment to surfaces [101, 102]. It is hypothesized that hydrophobins increase the hydrophobicity of soil aggregate surfaces, protecting them against the disruptive force of water and hence increasing aggregate stability [103] (Fig. 2). Rillig et al. [104] have demonstrated that the mycelium of AMF can effectively increase water repellency and the related stability of aggregates against water. Thus far no hydrophobins from AMF have been identified, although they—or very similar compounds—are expected to exist [29].

Within the context of fungal products, glomalin has received considerable attention in the last 20 years as a component that enhances adhesion of soil particles, thereby enhancing aggregate stability [105] (Fig. 3). At the moment, it is still only an operationally defined pool of SOM as glomalin-related soil protein (GRSP), due to a lack of a full biochemical description, not knowing who produces it and what it does in soils [60, 106]. Glomalin alone cannot be (easily) separated and extracted GRSP fractions—especially when using the commonly applied Bradford assay—have been shown to contain a large carbohydrate fraction as well as many other proteinaceous compounds; a mix of everything produced by fungi, bacteria, protists, and plants [105, 107, 108] but see Ref. [109]. Hence, a pure fungal origin could not be confirmed [110]. Nevertheless, multiple studies correlate higher GRSP concentrations to increased stability of soil aggregates [56, 111–114]. The mechanism(s) that contribute to this correlation are largely unstudied, but it is suggested GRSP could contribute to hydrophobicity and/or act as a gluing agent, a so-called “necroglue”. This term is used to describe the sticky nature of necromass and glomalin-like substances [105, 111].

We have only described potential mechanisms that can positively contribute to the formation and stabilization of soil aggregates so far. Overall, especially Basidiomycota have been shown to have neutral effects on soil aggregation [41], and generally, SF can also be expected to destabilize aggregates by breaking down cementing agents with their repertoire of degradative enzymes (Fig. 3) [115]. AMF can also stimulate saprotrophic microbes to decompose SOM by releasing exudates (i.e. priming) [116], freeing up N and P for acquisition [117]. Moreover, possibly in combination with enzymatic activity, the hyphae are suggested to have a tunnelling effect: penetrating aggregates at points of weakness and pores and opening up pathways for water, oxygen, and other soil biota [115, 118]. A destabilizing effect of AMF is not expected as they acquire carbon from their host plants [117], but other mycorrhizal types can produce degradative enzymes although in lower quantities than SF [29, 119]. Studies specifically targeting this potentially destabilizing effect do not exist, and it is possible the positive contributions of AMF to aggregation outweigh any negative effects.

### Biological interactions

The soil microbial community consists of fungi and bacteria, which are commonly linked to aggregation, but also archaea, algae, protozoa, and viruses (Fig. 3). Although these do not affect aggregation as directly as hyphae-growing fungi, they are part of the larger soil food web connecting all soil organisms [22]. Additionally, soil fauna of varying sizes (nematodes, mites, springtails, and earthworms) within this food web may feed on fungal hyphae or their products, as well as shape soil’s structure [73, 86, 94, 120, 121]. Fungal physiological responses to grazing include changes in hydrolytic enzyme production and respiration rates. These directly affect nutrient mineralization and the flux of CO_2_. Preferential grazing may also exert selective pressures on saprotrophic communities, driving shifts in fungal succession and community composition (Fig. 3). High-intensity grazing often reduces fungal growth and activity, whereas low-intensity grazing can have stimulatory effects [25, 122]. Invertebrate diversity and community composition represent key factors determining the functioning of fungal communities and the services they provide, most likely also soil aggregation (Fig. 3).

### Variation in contribution to aggregation between and within fungal species

The aforementioned mechanisms are related to certain fungal characteristics, such as mycelial architecture, and the amount and quality of gluing agents produced. The expression of these characteristics will vary from species to species, and hence, the effect on soil (macro)aggregation is expected to be species-specific [123–125]. Studies that specifically target and compare the ability of various fungal species to contribute to aggregation are rare. Schreiner et al. [69] found more large (2–4 mm) water-stable aggregates when pots with soybean were inoculated with *Glomus mosseae* as compared to two other AMF species. In the meta-analysis by Leifheit et al. [84], only *G. mosseae* and *Glomus intraradices* were well enough represented among included studies to allow comparison and both showed a similar positive contribution to soil aggregation. For SF, Lehmann et al. [85] showed some variation both between and within three major taxonomic groups, with species of the phylum Ascomycota generally having the best aggregate-forming capability. This study was only the first to infer what measurable fungal properties make certain species good aggregators. Nonetheless, although data are still limited, Ascomycota, Glomeromycota, and Mucoromycotina have on average similar and slightly positive effects on aggregation. Tested isolates within the phyla Basidiomycota have no significant negative or positive effect [73]. However, all studies have shown large variation in aggregate-forming capability across species and this is likely a characteristic not conserved in phylogeny [85, 89].

Within fungal species differences occur in expression of characteristics related to soil aggregation Fig. 3; [69, 125]. As for between species, this can be caused by inherent genetic variation, but also can be explained by phenotypic plasticity regulated by biotic and abiotic controls [124]. In the case of AMF their host plant is an important control for their overall functioning and hence their potential contribution to increased stability of aggregates [70]. This suggests an AMF “aggregation specialist” may not exist or is at least very much context-dependent [24]. We are only beginning to learn which fungal traits are relevant for soil aggregation [85, 115, 124, 126] and have little understanding of how these traits vary in response to variation in abiotic (soil texture and structure, pH, nutrient availability, and moisture) and biotic (interactions with plant roots, other fungi, and bacteria) factors.

### Feedback mechanisms between soil structure and fungi

Only direct effects of filamentous fungi on soil aggregation are discussed thus far, but we cannot dismiss the existence of feedback loops between the two [24, 42]. By contributing to change in the size and shape of aggregates, fungi also alter the intra- and interaggregate pore space geometry and consequently the transport and availability of, for instance, nutrients and moisture [127]. Pore space is the habitat of soil organisms—in large part defined by the properties of aggregates—and hence should not be omitted when regarding the role of fungi in soil aggregation. The size of the pores between aggregates and the connectivity of these pores influence their aeration and hydrology. Thus, soil organisms essentially change or reinforce their own habitat—and that of other soil organisms—which may have consequences for the species composition and activity of fungi [91]. The relationship between soil structure and microbial activity has so far only received limited attention in literature, especially in quantitative approaches [42, 128]. Although it is likely feedback mechanisms exist, no studies are available to make any predictions about their relative importance for aggregate formation and turnover.

### Importance of fungal processes in different environments

Besides differences in fungal characteristics, the relative (overall) contribution of soil fungi to aggregation varies with several factors related to the local environment. Conditions can be unfavourable for fungal growth, and hence, their contribution to soil aggregation is limited (soil management; Fig. 3). Extreme conditions (very low/high pH, metal toxicity, waterlogging, and drought) usually not only preclude fungal activity but also biomass production and soil aggregation in general. Absolute fungal abundance decreases with soil depth likely because of the limited and heterogeneous availability of organic matter and oxygen, making physical processes more important in subsoils [129, 130]. Certain agricultural practices (such as application of fungicides or frequent tillage) can also strongly reduce fungal activity. Physical disruptions of the soil negatively impact filamentous fungi as their hyphal network can be destroyed, and mycorrhizal fungi lose their viable plant host in the case of harvest, especially when followed by a fallow period [131, 132]. Moreover, filamentous fungi likely experience a competitive disadvantage following redistribution of moisture and nutrients compared to single-celled organisms such as many bacteria [57].

The relative importance of fungi depends on various other soil components and processes that also control soil aggregation. In some cases, the role of fungi will be negligible, whereas in others the presence of fungi can be crucial for aggregate formation and stabilization. Any biotic influences on aggregation are expected to be strongly reduced in soils with a high clay content (ca. >35%), because drying–wetting cycles will be the main driver, e.g. in presence of 2:1 phyllosilicates with a large shrink–swell capacity [58]. Also, soils high in metal oxides have mainly abiotic-mediated (micro)aggregation [59, 94], and these soils cannot be described by the aggregate hierarchy model as OM is not the main binding agent [86]. Unfortunately, abiotic factors (soil texture, clay mineralogy, and pH) and biotic factors (fungal activity, relative contribution of fungi to total biomass, fungal diversity, and root architecture) are rarely studied together within the context of soil aggregation, and hence, quantitative data are scarce [29]. Field studies are mostly focused on the effects of varying management practices on aggregation and fungi, usually limited to topsoils [56, 132]. Moreover, in experimental (pot) studies conditions are often chosen such that the expected effect is the largest, excluding most other abiotic and biotic factors to establish causality [29]. Although necessary, this raises questions about the relative importance and functioning of fungi in real ecological settings. We are therefore in need of studies that integrate both the biotic and abiotic factors on soil aggregation in intact soil columns.

## Integration of fungal community characteristics to understand soil aggregate formation

The ultimate outcome of studying the interaction between fungi and soil aggregation is a numerical model that includes the most relevant abiotic and biotic factors across the sites to represent the mechanisms of fungal-mediated aggregation and successfully predict its responses to management and environmental changes. This could then be integrated with general models of soil aggregation and SOC dynamics (Box 1). As true mechanistic understanding is still missing, no detailed numerical models of fungal-mediated aggregation currently exist [29, 42, 130]. We focus on discussing how soil structure and fungi could be quantified in ways meaningful for modelling aggregate and SOC dynamics. In Figs 1–3, frameworks for SOC dynamics, soil aggregate turnover, and fungal-mediated aggregation are presented, summarizing the current understanding of the processes, drivers, and interactions involved. These could be used as the theoretical backbone of future models, but such a visualization will also help identifying research needs and formulating new research questions and hypotheses.

**Box 1 Including aggregate formation theories in soil carbon modelling** 

The need for SOC models operating on smaller spatiotemporal scales, including measurable C pools, and new insights into the key processes behind SOC dynamics have stimulated the formation of new numerical models [11, 126, 127]. Most of these models focus on microbial activity and substrate availability, ignoring the smaller but potentially still important effects of molecular structure on degradability and stability of formed composite structures [128]. Knowledge and data of aggregate turnover rates are lacking [126], especially for microaggregates, and usually only weight-based indices such as water-stable aggregates (WSA) or mean weight diameter (MWD) are measured in laboratory and field studies. Turnover rates are very hard to determine, as it requires non-disruptive sampling and measuring techniques, and potentially long time series. Promising new and advanced methods such as labelling with rare earth elements and stable isotopes, coupled to visualization techniques such as X-ray computer tomography, offer new insights into aggregate and pore space dynamics [156, 158–161], although translation of results to the field remains challenging.

For most experimental studies on fungal-mediated soil aggregation, the application of, for instance, rare earth elements will not be feasible (too expensive and time-consuming) and aggregation cannot be quantified as turnover rate. X-ray tomography or time-lapse series of the soil matrix assessed by confocal laser scanning microscopy might be able to quantify aggregate turnover rates in the near future [129]. Research focusing on linking aggregate size distributions to aggregate turnover/breakdown rates under various conditions is highly desirable to make WSA/MWD data more useful. Microaggregates-within-macroaggregates has been proposed as indicator to track changes in SOC stabilization (capacity), in particular for agricultural soils, building on the notion that C is preferentially stabilized in microaggregates occluded in macroaggregates with slow turnover, i.e. water-stable macroaggregates [129–131]. Microaggregates within water-stable macroaggregates are usually not isolated and reported, but this should be done in future studies in a wide variety of settings to test its viability as a diagnostic fraction for SOC stabilization.

However, including microbial spatial effects into global carbon models is not yet possible due to the high variation and the very small scale these processes take place [129]. Aggregates or a volume of aggregates could be used as an intermediate scale to model microbial contributions to global carbon models. Especially in earth system models that integrate physical, chemical, and biological processes occurring on land, the oceans, and the atmosphere adding microbial effects on aggregate scale might be a valuable angle in this [129]. Due to the increase in computational power microbial attributes, such as carbon use efficiency and growth efficiency of microbes can be included, this paves the way for a new generation of global carbon models that model these processes from the aggregate to global level.

### Measuring fungal activity

For the quantification of soil fungi in relation to soil aggregation two aspects are important: function and activity. In many incubation studies, macroaggregation increases shortly after addition of fresh SOM and declines a few weeks after the last addition [50, 51, 133, 134]. The common explanation is that the microbial community will have consumed most of the SOM, and subsequently, fungal activity will be strongly reduced when its primary energy source is depleted. Mycelia will not further extend and/or not produce gluing agents to support the formation and stabilization of new aggregates (Table 1; Figs 2 and 3). Moreover, most hyphae are said to be only short-lived in soil, with an average lifespan ranging from days to at most months [135–138], weakening the stability of existing (macro)aggregates during turnover. However, at the same time the created necromass provides a new source of stabile carbon itself [139, 140]. This highlights the importance of a constant above and below ground input of new organic materials for soil aggregation. Hence, fungal activity and turnover (lifespan) are critical features for any soil aggregation model. Formation may be proportional to the SOM addition rate and may vary with chemical composition as well as soil conditions and community composition [50, 51, 141]. Determining fungal activity in soil directly, especially in the form of hyphal extension rate, is very challenging (Table 1). Activity may be inferred from soil respiration rates, proxies for fungal biomass (ergosterol, PLFA/NLFA), enzyme production, RNA, or substrate incorporation rates, often using isotope-labelled materials traced with DNA- or RNA-(q)SIP or in NanoSIMS [21, 142–146], and/or measurements on past active biomass by measuring necromass using amino sugars [147]. Nevertheless, fungal activity alone in the form of biomass production is not enough, because it is the investment in mycelial architecture (density), hyphal structural integrity and persistence, and enzymes and other exudates that likely matter for soil aggregation (Table 1) [85, 89, 115].

A trait-based approach has only recently been proposed for fungal-mediated aggregation research (Box 2) [115, 124], and hence, data are lacking to confirm the proposed fungal traits (Table 1) as important for aggregation or demonstrate trait variability between species and environmental settings (but see Zheng et al. [148]; Lehmann et al. [85, 149]). Lehmann et al. [85] have been the first to demonstrate that studying aggregation-related traits of SF is possible. The biomass density of individual fungal species measured on agar plates explained aggregation in artificial systems. They focused on SAF, and not stabilization and disintegration of aggregates which require slightly different approaches [115]. However, we think for future research it will be important to include traits that relate to potential particle adhesion to hyphae or any production of gluing agents, because no measures or proxies have been developed yet on these characteristics. On top of this, the effects of grazing and other interactions on SF should be investigated more [25]. In the next two sections, we summarize developments needed to successfully quantify fungal function, addressing a number of limitations, complemented by recent advances and alternatives to the trait-based approach.

**Box 2 Measuring fungal function: a trait-based approach for better mechanistic understanding** 

Function refers to the ability of fungi to positively or negatively contribute to soil aggregation via the proposed mechanisms (enmeshment, gluing agents, and degradative enzymes; Table 1 and Fig. 3). The fungal characteristics connected to this ability can be quantified by converting them to functional traits, i.e. defined and measurable properties that can be attributed on the individual level to enable comparison between and within species under varying environmental conditions [47, 97, 145]. For instance, the ability to enmesh particles will likely be related to mycelial architecture/morphology, which can be measured as hyphal length and extension rate, biomass density, and hyphal branching angle (Table 1) [59, 146, 147]. The application of fungal traits, and a better knowledge about fungal life-history traits in general, will mostly be useful to increase the mechanistic understanding of fungal-mediated aggregation, corroborating the currently hypothesized mechanisms with causal relationships instead of anecdotal evidence.

Trait data are also used to better understand how natural communities are organized and vary in structure across time and space, aiming to find general patterns and rules in community ecology [103, 145, 148]. This can ultimately be useful in discerning variation and changes in soil aggregation via, for instance, differing species compositions and interactions. It is up for discussion whether a trait-based approach is currently also of use for modelling soil aggregation and SOC dynamics, and the concomitant implementation of land management strategies. In the next three sections, we summarize three developments needed to successfully quantify fungal function, addressing a number of limitations, complemented by recent advances and alternatives to the trait-based approach.

Experimental research of mycorrhizal fungi is challenging as they depend on a plant host and thus usually require a complex soil matrix, plus involve labour-intensive steps to isolate (intact) mycelia from soil and root systems [146]. Systematic studies, i.e. Ref. [59], on traits of mycorrhizal fungi saprotrophic fungi and plant roots in relation to aggregation do not exist, or are limited to non-specific measures like biomass and root colonization [102]. On top of the soil physicochemical conditions, the host plant species will determine to some degree the observed fungal trait values. Initially, a diverse yet small number of plant species should be studied across research groups to enable easy comparison and establish key traits and mechanisms for both fungi and plant roots. Not only fungal traits measurements but also the methodology of soil aggregation analyses should be standardized. Furthermore, to allow for cross-study comparisons, a full physicochemical description of the used soil (field and experimental conditions) should be reported and included in the metadata of database entries. Alternatively, the international use of a standardized artificial soil or standardized soil mixture coming from one location could be considered “assays” [47].

**Table 2:** Identified knowledge gaps (in bold) that currently limit the quantification of the relationship between (filamentous) fungi and soil aggregation in function of soil organic carbon (SOC) dynamics, with their respective research needs to close these gaps.

(1) **Quantification of soil aggregation in ways meaningful for SOC dynamics** (1.1) Determination of aggregate turnover rates across various environmental settings, using a combination of (advanced) methods [133, 164] (1.2) Estimation of aggregate turnover rates by using data from (1.1) linked to common aggregation indicators (e.g., WSA, MWD), potentially in combination with several abiotic and biotic factors (1.3) Development and implementation of microaggregates-within-macroaggregates as diagnostic fraction, possibly extended with aggregated mineral-associated organic matter (MAOM) [66, 165] (1.4) Improved fundamental understanding of microaggregate formation, stabilization and disintegration, elucidating the relative importance of various abiotic and biotic factors and processes by applying combination of advanced techniques (e.g., imaging, modelling) [42] (1.5) Integration of the soil aggregation and pore space perspectives [17, 66]
(2) **Confirmation of hypothesized biophysical, biochemical and biological mechanisms of fungal-mediated soil aggregation** (2.1) Adopting a trait-based approach to reveal measurable fungal properties positively or negatively impacting formation, stabilization and/or disintegration of macro- and/or microaggregates [115, 124] (2.2) Development of direct or indirect measures of fungal traits, especially in relation to particle adhesion and production of gluing agents (2.3) Development of a standardized methodology to study fungal traits in the lab and field [85] (2.4) More complex pot/culture studies that include biotic interactions to study biological mechanisms [29] (2.5) Application of novel techniques such as advanced imaging to study the effect of fungi on aggregate turnover in 4D (integration with 1.1) [29] (2.6) Investigating the effects of soil aggregates (and intra- and interaggregate pore spaces) on fungi (potential feedback mechanisms) [24, 128]
(3) **Quantification of the relative importance of fungi in the formation and turnover of soil aggregates across various settings** (3.1) Development of relevant indicators (measurable in field samples) more specific for fungal function related to soil aggregation (e.g., hyphal length vs. hyphal biomass density; glomalin), learning from (2) (3.2) Field studies of soil aggregation covering environmental gradients including abiotic and biotic factors, including (3.1) and also representing other biota than fungi [14, 81] (3.3) Longer (> year) and more complex experimental pot and field studies, especially including abiotic processes (e.g., freeze-thaw cycles), to improve translation of results to field conditions [29, 84]
(4) **Better representation of (measures of) fungal diversity in research on soil aggregation and SOC cycling** (4.1) Field and pot studies on soil aggregation including gradients of fungal diversity, potentially combined with variation in plant species or other soil organisms, and some key abiotic factors [24, 84] (4.2) Increased attention for saprobic fungi in fungal-mediated soil aggregation research [115] (4.3) Development of approaches to quantify fungal diversity that relate to their activity and function in relation to soil aggregation, potentially also integrating other functions (e.g., C cycling) or full soil biodiversity [22]
(5) **More effective communication and data exchange among researchers of fungi and soil aggregation** (5.1) Public and curated database(s) for fungal functional traits related to soil aggregation, including relevant meta-data on (soil) physicochemical conditions and (potentially) biotic interactions, in support of (2) [115, 150] (5.2) Public and curated database(s) for field and pot studies covering soil aggregation and gradients of abiotic factors and biological diversity, highlighting data gaps and promoting common methodologies (5.3) Improved taxonomic, phylogenetic, biogeographic and ecological description of fungal taxonomic units (stored in linkable public databases), allowing interpretation and extrapolation of results [150] (5.4) More attention for and inclusion of culturable species to improve fungal taxonomic research [151]

WSA: water-stable aggregates; MWD: mean weight diameter

### Public databases and a common fungal taxonomy

Critical for the success of a trait-based approach will be the use of public databases and a consistent fungal taxonomy to enable effective communication among scientists. To support the rapidly growing field of fungal functional ecology, a new database for fungal traits (FunFun) has been made for new and published datasets [150]. But also FungalTraits [151] and FunGuild [152] already contain very useful information to link fungal species to functions. Researchers studying fungal-mediated aggregation should also access and share trait data here, benefit from datasets covering the same traits but collected even though studying different fungal functions (water and nutrient uptake and carbon and nitrogen cycling), and perhaps look for previously unknown trait associations that may be (indirectly) related to soil aggregation. This fulfils at least in part the need, or provides a framework, for a fungal traits database in aggregation research as described by Lehmann and Rillig [115]. A limiting factor for fungal taxonomy will be that currently only a small part of the large (estimated) number of fungal species has been named or described [150] (and references therein). Recent advances in DNA sequencing, especially high-throughput sequencing, have greatly increased the number of observed fungal species in environmental samples, but is challenged by accurate delineation of species and taxonomic assignment [72, 153]. The advice is to culture fungi and measure their traits more and not only sequence, which is a shift in thinking across the field. Extending our knowledge on culturable fungi by including more species and linkable ecological (trait) data is needed to improve fungal taxonomy as well as phylogenetic, biogeographical, and ecological interpretation of data [150, 154].

### Fungal diversity as proxy for overall fungal functioning

We are currently at the stage of establishing key fungal traits and methods to measure them, but we should soon also start including ecological realism to fully understand variation in fungal-mediated aggregation and SOC stabilization. In this case, a trait-based approach may not be fruitful at first. Especially, the large (possible) number of biotic interactions observed in the field cannot be reproduced in the laboratory, whereas in field studies, individual traits are very difficult to measure and only community averages can be determined at best. A potential solution is to look at fungal diversity as an integrative proxy for function, based on the assumption that with a larger (taxonomic, phylogenetic) diversity also a more diverse set of (complementary) traits will likely be present, maximizing total function [155]. This may be extended to include other groups of the soil community as well as plants for their (interacting) root systems. However, observed fungal diversity should be corrected for activity, as species may be dormant (only present as spores), (temporarily) relatively inactive compared to others, or only present as necromass [144]. Of course there are also ecosystems where bacteria are more important in terms of activity, such as in intensively managed croplands. Hence, the actual active functional diversity may be lower than expected based on plain diversity values. Fungal diversity is hypothesized to be positively correlated with water stable aggregates (WSA), but these are rarely studied together [24, 156, 157]. Leifheit et al. [84] found no support for this relationship in their meta-analysis, but this may be a remnant of highly differing approaches in studies included. We need more laboratory as well as field studies with gradients of fungal diversity also measuring soil aggregation, preferably in conjunction with abiotic factors (texture and clay minerology) and diversity of other biota (plants and (rhizo)bacteria). We will need additional experiments in which fungal diversity is manipulated by either removal by sieving or dilution, or added by using synthetic communities of cultured fungi. Observed species compositions can serve as basis for more targeted research into traits of certain species or reveal whether composition matters at all for soil aggregation in the field.

## Future directions and conclusions

In this review paper, we have summarized the current knowledge of fungal-mediated soil aggregation and discussed the most important knowledge gaps that limit our ability to quantify this process in relation to SOC dynamics. In Table 2, we have listed the five identified knowledge gaps and collated them with their respective research needs to resolve the gaps. Filamentous fungi can play a key role in the formation and stabilization of soil aggregates, which can protect incorporated SOM from decomposition by soil organisms. The turnover rates of macroaggregates and especially microaggregates determine the degree of protection, but very limited quantitative data are available for true mechanistic understanding and implementation of aggregate turnover in SOC modelling (Box 1) (1). The interacting biophysical, biochemical, and biological mechanisms of fungal-mediated soil aggregation, such as hyphal enmeshment and provisioning of gluing agents, are currently just hypotheses (2). Adopting a trait-based approach shows promise to increase the level of mechanistic understanding, but will require a coordinated effort of research groups and the development of standardized methodologies (Box 2). We also need field studies and more complex mesocosm studies that include both abiotic and biotic factors to elucidate the overall relative importance of fungi across environmental settings that are ecologically complex such as experiment that manipulate diversity by sieving, diluting, or addition of cultured fungi (3). The interactive relationship between fungal diversity (phylogenetic and functional) and soil structure is currently underrepresented in research, whereas diversity and species compositions are expected to be important for aggregation and SOC dynamics (4). We need a more effective communication and more collaboration among a diverse set of soil scientists and (fungal) ecologists, which will be greatly helped identifying more fungal species and improving the databases (traits and phylogeny) and soil aggregation (5). Overall, this all highlights the necessity to further integrate (soil) ecology with dominant physical and chemical perspectives if we want to fully understand the underlying processes of SOC stabilization across spatiotemporal scales. Only then can we make accurate numerical models for soil aggregation and SOC cycling and successfully predict the effects of land management strategies and global change.

## Data Availability

Data sharing not applicable to this article as no datasets were generated or analysed during the current study.
